# Comparison of ultrasound-guided transversalis fascia plane block and anterior quadratus lumborum block in patients undergoing caesarean delivery: a randomized study

**DOI:** 10.1186/s12871-023-02206-w

**Published:** 2023-07-21

**Authors:** Sezgin Bilgin, Hakan Aygun, Caner Genc, Burhan Dost, Serkan Tulgar, Cengiz Kaya, Nezih Sertoz, Ersin Koksal

**Affiliations:** 1 Department of Anesthesiology and Reanimation, Faculty of Medicine, Ondokuz Mayis University, Samsun, Turkey TR55139; 2 Department of Anesthesiology, Cigli Regional Training Hospital, Izmir, Turkey; 3 Department of Anesthesiology and Reanimation, Samsun Training and Research Hospital, Samsun, Turkey; 4 Department of Anesthesiology and Reanimation, Faculty of Medicine, Samsun Training and Research Hospital, Samsun University, Samsun, Turkey; 5 Department of Anesthesiology and Reanimation, Faculty of Medicine, Ege University, İzmir, Turkey

**Keywords:** Anesthesia, Analgesia, Analgesics, Cesarean section, Obstetrical, Opioid, Nerve block, Pain management, Postoperative, Ultrasonography

## Abstract

**Background:**

Cesarean section is becoming increasingly common. Well-managed postoperative analgesia improves patient comfort while encouraging early ambulation and breastfeeding. The analgesic efficacy of transversalis facial plane block (TFPB) vs. anterior quadratus lumborum block (QLB) was compared in this study.

**Methods:**

We analyzed the data of 49 pregnant women (gestation, ≥ 37weeks; age, 18–45years) scheduled for elective cesarean delivery (CD) under general anesthesia. They were randomly divided into TFPB and anterior QLB groups. All blocks were administered bilaterally with 25mL of 0.25% bupivacaine under ultrasound guidance prior to extubation. Postoperative morphine consumption and numerical rating scale (NRS) pain scores (static and dynamic [during coughing]) were recorded at 1, 3, 6, 9, 12, 18, and 24h.

**Results:**

There was no difference in postoperative morphine consumption between the groups at the third, sixth, and ninth hours, but the anterior QLB group consumed less morphine at the 12th, 18th, and 24th hours. Except for the first hour, resting and dynamic NRS scores were comparable between the groups. The first-hour resting and dynamic NRS scores were lower in the TFPB group (resting NRS, anterior QLB group, median [interquartile range], 2 [2–3] vs. TFPB group, 2 [0–2], *p* = 0.046; dynamic NRS, anterior QLB group, median [interquartile range], 3 [2–4] vs. TFPB group 2 [0–3], *p* = 0.001).

**Conclusions:**

In patients undergoing CD, anterior QLB decreased morphine consumption in the late period (9–24h) compared to TFPB, while pain scores were similar between both groups. The reduction in morphine consumption was statistically significant, but not clinically significant.

## Introduction

Since the introduction of ultrasound technology in anesthesia practice, interfacial plane blocks have become a part of postoperative analgesia management for many surgical procedures, including cesarean delivery (CD) [1]. The transversus abdominis plane (TAP) block has been used as part of postoperative multimodal analgesia in CD for many years, especially when intrathecal morphine is not available [2–4]. Ilioinguinal-iliohypogastric block, quadratus lumborum block (QLB), erector spinae plane block, and transversal fascia plane block (TFPB) can be used instead of TAP block for analgesia in lower abdominal surgeries such as CD [5–8].

The anterior QLB, described by Børglum et al. [9], is recommended for postoperative analgesia in abdominal surgeries [10]. The anterior QLB is deeper in the target facial plane than the lateral and posterior QLB, and in close proximity to internal organs, thus it is a technically challenging block [11, 12]. The TFPB is a more superficial fascial plane block than the anterior QLB and is used for analgesia in CD [7, 13]. In the literature, anterior QLB and TFPB are compared only in the context of inguinal hernia repair surgery, whereas they are compared with different block types separately for CD cases [10, 14–16]. To the best of our knowledge, the present study is the first to compare the effectiveness of these two blocks for patients undergoing CD. This study aimed to compare the morphine consumption and pain scores of patients who received bilateral anterior QLB and TFPB performed at the end of CD surgery with general anesthesia in the first 24 h postoperatively.

## Methods

### Study protocol

This was a single-center, prospective, randomized (1:1) controlled, double-blind, parallel group study. The study was approved by the local ethics committee (OMU-KAEK 2021/379) and Ministry of Health (2021-AKD-764205) and registered on ClinicalTrials.gov prior to the initial patient recruitment with registration number NCT05408403. The manuscript was written in accordance with the CONSORT guidelines.

### Participants

The study was conducted at a training hospital between June and October 2022. Written informed consent was obtained from all participants for the interventions before including the study. The study included patients aged 18–45 years, with an American Society of Anesthesiologists (ASA) score of II and gestational age of ≥ 37weeks, scheduled for elective cesarean section via a Pfannenstiel incision under general anesthesia. Patients with severe renal, cardiac, hepatic disease; those requiring spinal anesthesia; obese patients (> 100kg, BMI > 35kg/m^2^); and patients with contraindications to interfascial plane blocks (severe coagulopathy, infection at the injection site etc.), hypersensitivity to local anesthetics, or history of allergy were excluded from the study. Patients with a history of opioid use for more than 4 weeks, psychiatric disorders, and anatomical deformities and those who refused to participate were also excluded. And patients with pre-eclampsia, eclampsia, percreata, and accreata complications, and massive obstetric hemorrhage were excluded from the study.

### Randomization and blinding

The patients were divided into two groups of 25 patients each. The sealed envelope technique was used for randomization. All patients were assigned a randomization ID. This ID was used during postoperative follow-up. An experienced anesthesiologist who would not be involved in the patient's intraoperative or postoperative care and would only perform the block procedure opened the sealed envelope 1h before the surgery to learn which group the patient would be assigned to. Intraoperative and postoperative follow-up examinations were performed by two different physicians who were blinded to the patient group.

### Anesthesia management

All patients were administered general anesthesia according to our clinic's standard CD protocol. No premedication was administered to the patients. In addition, all the patients were started on intravenous infusion of Ringer’s lactate solution (5–7mL/kg/h). After ASA-recommended standard monitoring (non-invasive blood pressure monitoring, electrocardiography, and peripheral oxygen saturation), anesthesia was induced with propofol (2.5mg/kg) and rocuronium (0.6mg/kg), followed by tracheal intubation. After the umbilical cord was clamped, remifentanil was administered at 0.25mcg/kg/min as an analgesic. Sevoflurane and O_2_-air were administered to maintain general anesthesia (inspired oxygen fraction, 0.40). For volatile anesthetic maintenance, a minimum alveolar concentration (MAC) of 1, determined by age, was maintained until placenta clamping, at which point a MAC of 0.5–0.75 was maintained. The rate of remifentanil infusion was adjusted according to hemodynamic parameters. Extubation was performed at the end of the operation after neuromuscular recovery was achieved with 0.04mg/kg neostigmine and 0.02mg/kg intravenous (IV) atropine. The surgical team did not administer infiltrative analgesia. The patients were routinely administered ondansetron (4mg IV), approximately 20min before extubation to prevent postoperative nausea and vomiting.

### Interventions

All ultrasound-guided fascial plane blocks were performed before extubation at the end of surgery, in accordance with the rules of asepsis/antisepsis. In both blocks, a low-frequency convex transducer (2–5MHz, LOGIQ V1, GE Healthcare, USA) and block needles (21 G, 100mm, SonoPlex STIM Pajunk, Germany) were used. As a local anesthetic agent, 25mL of 0.25% bupivacaine (Marcaine®, Astra Zeneca, US) was used bilaterally.

### Transverse fascia plane block (TFPB)

**Fig. 1:**
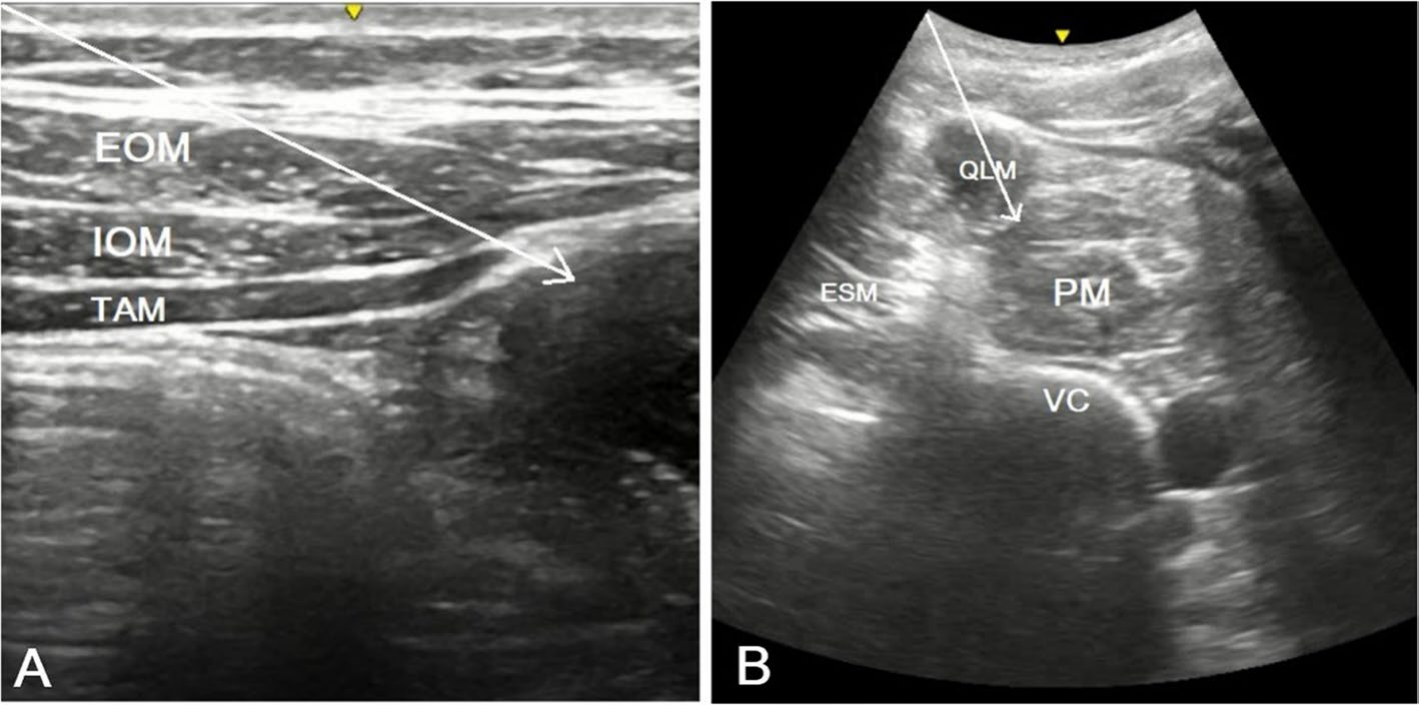
**A–B** Relevant sonoanatomy for US-guided TFPB and Anterior QLB. (A) US-guided TFPB. The white line represents the needle trajectory. (B) US-guided anterior QLB. The white line represents the needle trajectory. TFPB, transversalis fascia plane block; EOM, external oblique muscle; IOM, internal oblique muscle; TAM, transversus abdominis muscle; QLM, quadratus lumborum muscle; PM, psoas muscle; ESM, erector spinae muscle; VC, vertebrae corpus

With the patients in the supine position, the transducer was first placed transversely, just above the iliac crest, and slightly tilted caudally. The skin, subcutaneous fat, external oblique muscle, internal oblique muscle, transversus abdominis muscle, endpoint, transversalis fascia, retroperitoneal adipose tissue, and peritoneum were all visible (Fig. 1A). Using the in-plane technique, the needle was advanced to the endpoint of the transversus abdominis muscle. After 3mL of saline was used to confirm the facial plane, a local anesthetic was administered as soon as the deep fascia of the transversus abdominis muscle was passed. The movement of the retroperitoneal adipose tissues to the deep plane was visualized. The same process was applied to the other side.

### Anterior QLB

While the patient was in the lateral decubitus position, the transducer was first placed between the iliac crest and subcostal margin. The abdominal muscles, latissimus dorsi muscle, erector spinae muscle, psoas muscle, transverse process of the 4th lumbar vertebra, and vertebral corpus were visualized by sonography (Fig. 1B). In the facial plane, a 21-gauge, 10-cm long needle was advanced in the plane between the quadratus lumborum (QL) and psoas muscles. After the administration of 3mL saline to confirm the facial plan, 25mL of 0.25% bupivacaine was administered. The procedure was repeated on the other side.

### Postoperative management

All patients received 1g of IV paracetamol 30min before surgery and another 1g every 8 h in the hospital. A numeric rating scale (NRS) was used to measure the pain level. Patients were informed about the NRS scoring system (0 points, indicating no pain; 10 points, indicating the worst pain imaginable) during the preoperative period, and if their NRS score at rest was greater than 3, they were informed that they could request painkillers from the patient-controlled analgesia device (PCA). Patients were monitored in the post-anesthesia care unit after extubation. Both groups of patients received an IV-PCA (Bodyguard 575 pain manager, UK) device containing 0.5–1mg/mL of morphine. The PCA settings were adjusted to 1-mg morphine bolus, 8-min lock time, and 24mg 4h limit time.

### Outcomes

The primary outcome of this study was the amount of opioid consumption in the first 24h after surgery; postoperative pain scores and the time of first opioid demand were the secondary outcomes. Morphine consumption was measured at 3, 6, 9, 12, 18, and 24h, and static and dynamic pain scores were measured at 1, 3, 6, 9, 12, 18, and 24h postoperatively.

A five-stage verbal descriptive scale (0 = absent, 1 = mild nausea, 2 = moderate nausea, 3 = vomiting once, and 4 = vomiting more than once) was used to score the intensity of nausea and vomiting. Ondansetron (4mg IV) was administered to patients with a score of ≥ 3. Reports of nausea and vomiting as well as technical and drug-related issues (respiratory depression, local anesthetic toxicity, hematoma, and organ damage) were recorded.

### Sample size

The mean 24-h cumulative morphine consumption in the pilot study, which included ten patients, was 6.08 ± 2.17mg in the anterior QLB group and 8.50 ± 2.55mg in the TFPB group. Therefore, with 95% confidence (1 − α), 95% test power (1 − β), and effect size d = 1.032, the sample size calculation determined that a minimum of 22 patients in each group should be included in the study. Given the risk of data loss, each group was designed with 25 patients, for a total of 50 patients.

### Statistical analysis

Statistical analyses were performed using IBM SPSS V23.0 (IBM, New York, USA). Normality was tested using the Shapiro–Wilk test. The mean ± standard deviation and median were used to express the continuous variables (25th–75^th^ percentiles). The independent samples t-test was used to analyze continuous variables with homogeneous variances. The Mann–Whitney U-test was used for data that did not show a normal distribution. The χ^2^ test was used for the comparison of ratios. Fisher's exact test was used to evaluate categorical variables (ASA classification, sex, and so on). Statistical significance was set at *p* < 0.05.

## Results

**Fig. 2:**
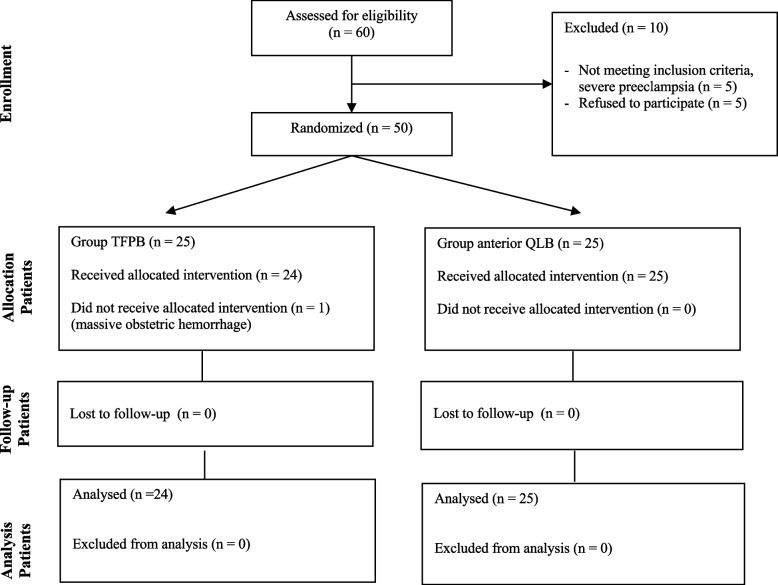
Flow diagram showing the distribution of patient data. Abbreviations: TFPB, transversalis fascia plane block; QLB, quadratus lumborum block

Sixty patients scheduled for elective cesarean section were screened for participation in the study. Ten patients were excluded from the study due to the following reasons: five patients were diagnosed with severe pre-eclampsia, and an additional five patients declined to participate. Therefore, 50 patients were included in the study. One patient with TFPB was excluded from the study because of massive obstetric hemorrhage. Figure 2 shows the flow diagram of our study. There were no differences in the demographic data between the groups (Table 1).

**Table 1:** Descriptive characteristics and first analgesic demand of patients

	**Group anterior QLB (*n* = 25)**	**Group TFPB (*n* = 24)**	**P**
Age (years)	25.58 ± 4.31	25.6 ± 3.84	0.986
Length (cm)	162 ± 5.2	163 ± 6.1	0.539
Weight (kg)	79.70 ± 4.79	80.2 ± 5.73	0.741
First analgesic demand time (hour)	3.70 ± 1.61	3.48 ± 1.58	0.631

Continuous variables are presented as mean ± standard deviation and categorical variables are presented as counts (percentages)

**Fig. 3:**
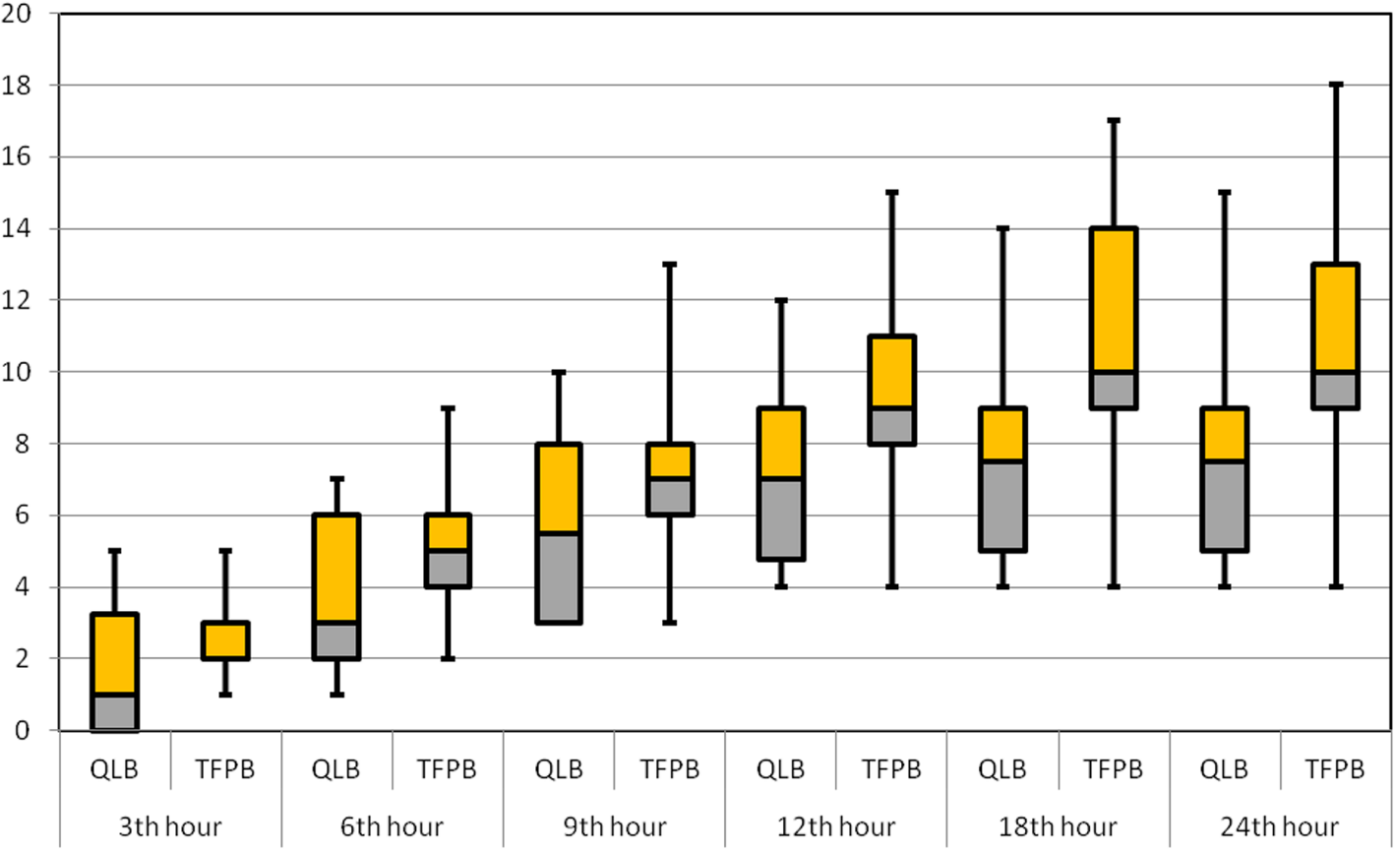
Cumulative postoperative morphine consumption of groups at different time-points (mg)

In the third, sixth, and ninth postoperative hours, there was no statistically significant difference between the groups in terms of morphine consumption (anterior QLB [median]; 1, 3, 5.5 vs. TFPB; 2, 5, 7 mg, respectively); however, at other time points, the anterior QLB group had statistically lower morphine consumption than the TFPB group (anterior QLB [median]; 7, 7.5, 7.5 vs. TFPB; 9, 10, 10 mg, respectively) (Table 2, Fig. 3). Furthermore, the initial opioid demand times of the two groups were comparable (Table 1).

**Table 2:** Cumulative morphine consumption in 24 h (mg), postoperatively

	**Group anterior QLB (*n* = 25)**	**Group TFPB (*n* = 24)**	**P**
3th hour	1 (0–3.25)	2 (2–3)	0.113
6th hour	3 (2–6)	5 (4–6)	0.066
9th hour	5.5 (3–8)	7 (6–8)	0.052
12th hour	7 (4.75–9)	9 (8–11)	**0.011**
18th hour	7.5 (5–9)	10 (9–13)	**0.002**
24th hour	7.5 (5–9)	10 (9–13)	**0.002**

Continuous variables are presented as median (interquartile range). Statistically significant differences are highlighted in bold

The resting and dynamic NRS scores were similar between the groups at all time points except for the first hour. The first-hour resting and dynamic NRS scores were lower in the TFPB group (resting NRS, anterior QLB group, 2 [2–3] vs. TFPB group, 2 [0–2], *p* = 0.046; dynamic NRS, anterior group QLB, 3 [2–4] vs. TFPB group, 2 [0–3], *p* = 0.001) (Table 3). Two patients in both the groups reported experiencing minor nausea. No block- or drug-related problems were observed.

**Table 3:** Static and dynamic NRS scores of patients

	**Group anterior QLB (*n* = 25)**	**Group TFPB (*n* = 24)**	**P**
Static NRS scores			
1st hour	2 (2–3)	2 (0–2)	**0.046**
3th hour	3 (2–4)	3 (2–4)	0.854
6th hour	4 (34)	4 (3–4)	0.870
9th hour	4 (3–4)	4 (3–4)	0.719
12th hour	3 (3–3)	3 (3–4)	0.054
18th hour	3 (2–3)	3 (2–3)	0.661
24th hour	1.5 (0–2)	2 (0–2)	0.315
Dynamic NRS scores			
1st hour	3 (2–4)	2 (0–3)	**0.001**
3th hour	3 (2–5)	4 (3–4)	0.587
6th hour	4 (4–5)	4 (3–5)	0.875
9th hour	4 (4–5)	4 (3–5)	0.712
12th hour	3.5 (3–4)	4 (3–4)	0.448
18th hour	3 (3–3)	3 (2–3)	0.668
24th hour	2 (0–2)	2 (0–2)	0.162

Continuous variables are presented as median (interquartile range). Statistically significant differences are highlighted in bold

## Discussion

In the present study, on the patients who underwent CD under general anesthesia, the total opioid demand in the first 24h was reduced in the anterior QLB group compared to that the TFPB group, but there was no difference in the early (0–9h) opioid requirements. The patients' postoperative resting and dynamic NRS ratings were similar, except for the first hour ratings.

There are two components of CD pain. The first is somatic pain from the skin incision, and the second is visceral pain from the exteriorization and straining of the uterus. The anterior branches of the T10–L1 (particularly T12–L1) spinal nerves should be blocked for somatic pain, and the superior/inferior hypogastric plexus branches should be inhibited for visceral pain [17, 18]. While neuraxial anesthesia/analgesia can relieve both types of pain[19], abdominal wall blocks are usually only effective for somatic pain [11].

TFPB selectively blocks the anterior branches of the T12 and L1 spinal nerves as well as the subcostal, ilioinguinal-iliohypogastric nerves. These nerves carry purely somatic innervations [7, 20]. Postoperative analgesia was achieved by iliac crest harvesting and inguinal hernia repair by blocking with TFPB, according to the literature [21–23]. On the other hand, local anesthetic applied to the facial plane spreads to the lower thoracic paravertebral area, providing both somatic and visceral analgesia in the anterior QLB [24]. According to some cadaveric studies, local anesthetic spread to the anterior QLB is limited to the L1–L3 nerve roots. There may be an alternative facial plane block that can be used to block the lumbar plexus [24, 25]. However, studies have suggested that the spread of the block to the T12–L1 is limited, and it may be a safe alternative for lower abdominal surgeries. Since there is no lumbar plexus spread, there will be no quadriceps weakness or ambulation issues [26]. When Børglum et al. first described the block, the dermatomal extension included T7–L1 and spread to the lower thoracic paravertebral area as a possible mechanism [9]. Despite inconsistent dermatomal extension results in cadaver studies, paravertebral spread of local anesthetic in the anterior QLB has been found to provide sufficient visceral and somatic blockade to provide postoperative analgesia in lower abdominal surgery [12].

In the present study, we predicted that anterior QLB would provide visceral analgesia in addition to somatic blockade and would have a better analgesic effect than TFPB, which only provides somatic analgesia. However, contrary to this prediction, the findings of this study revealed that the analgesic activities of both the blocks were comparable. The QL muscle runs in the craniomedial to the caudo-lateral direction as it progresses from the 12th rib to the ilium. The thoracolumbar fascia, latissimus dorsi muscle, lateral raphe, lumbar interfascial triangle, QL, and investing fascia form the lateral part of the paraspinal muscles below L2, while only the transversalis fascia forms the lateral part above L2. This anatomical difference has been reported to allow easy spread of local anesthetics to the posterior of the endothoracic fascia on the transversalis fascia and reach the lower thoracic paravertebral space through anterior QL injections, which are administered at levels higher than the L2 level [27]. Furthermore, injections close to the lateral arcuate ligament (L1–2) have been shown to have increased spread to the thoracic paravertebral space in some variations of the classical anterior QL [28–30]. As a result, injections from the L4 level in the classical anterior QL may prevent the local anesthetic from reaching the paravertebral area and providing adequate visceral analgesia.

A single study comparing these two blocks in lower abdominal surgery for inguinal hernia repair under general anesthesia was found in the literature; the postoperative analgesic activities of both blocks were comparable in this study [16]. In this study, anterior QLB seemed to be more effective, especially in the late period. However, CD is more comprehensive surgery than inguinal hernia repair; thus, the source of pain is more complex.

In our study, the pain levels were comparable at all measurement times (except for the first hour), and the cumulative morphine requirement was lower in the first 9 h (5.5 [3–8] mg vs. 7 [6–8] mg). We observed that these two blocks had comparable efficacy in the early postoperative hours, when post-surgical pain peaked. The anterior QLB appears to be superior in terms of opioid requirement in the long run (9–24h). However, this difference was small. In the anterior QLB group, the median opioid requirement in the 15h was 2 (IQR, 1–3) mg, and in the TFPB group, it was 3 (IQR, 3–4) mg. Recently, statistical and clinical significance debate has been raised when comparing opioid requirements in studies on fascial plane blocks [31, 32]. That is, such a slight difference means that these two blocks be considered clinically similar.

Quadriceps weakness, one of the most serious complications of these two blocks, has been reported after anterior QLB and TFPB [30, 33, 34]. However, given the anatomy, the anterior QLB may be more dangerous in terms of lumbar plexus spread. Since quadriceps strength was not assessed in our study, we cannot comment on motor weakness. The patients were placed in the lateral decubitus position for anterior QLB, whereas TFPB is usually performed in the supine position, although it can also be performed in the lateral position [7, 20]. The TFPB is used in the supine position and has a more superficial block, which may make it preferable [16].

CD is usually performed under neuraxial anesthesia in our clinic. Intrathecal morphine for postoperative analgesia is an effective and cost-effective method of analgesia [35]. We conducted our study with patients undergoing neuraxial techniques because the time to eliminate the effects of spinal analgesia cannot be standardized. However, the effectiveness of spinal anesthesia may be worth examining in subsequent studies, particularly in patients who receive intrathecal morphine [20].

Our study had the following limitations: first, dermatome examination could not be performed and there was no control group; second, block performance times, including time taken to position, were not recorded, patient ambulation and quadriceps strength were not evaluated.

## Conclusion

In the present study, the analgesic effects of the anterior QLB and TFPB blocks were found to be similar in the first 9h in patients undergoing CD under general anesthesia. However, there was a benefit to QLB in terms of reducing morphine consumption in the late period (9–24h), which was not clinically significant, but statistically significant. Both techniques improve the quality of the postoperative analgesia regimen when used in conjunction with multimodal analgesia in patients undergoing CD under general anesthesia.

## Data Availability

The datasets used and/or analysed during the current study available from the corresponding author on reasonable request.

## Abbreviations

TFPB: Transversalis facial plane block
QLB: Quadratus Lumborum Block
CD: Cesarean Delivery
NRS: Numeric Rating Scale
TAP: Transversus Abdominis Plane
ASA: American Society of Anesthesiologists
PCA: Patient-Controlled Analgesia
